# Antitumor Effects of *Cannabis sativa* Bioactive Compounds on Colorectal Carcinogenesis

**DOI:** 10.3390/biom13050764

**Published:** 2023-04-28

**Authors:** Rita Silva-Reis, Artur M. S. Silva, Paula A. Oliveira, Susana M. Cardoso

**Affiliations:** 1 LAQV-REQUIMTE, Department of Chemistry, University of Aveiro, 3810-193 Aveiro, Portugal; reis.rita@ua.pt (R.S.-R.); artur.silva@ua.pt (A.M.S.S.); 2 Centre for the Research and Technology of Agro-Environmental and Biological Sciences (CITAB), Inov4Agro, University of Trás-os-Montes and Alto Douro, 5000-801 Vila Real, Portugal; pamo@utad.pt; 3 Clinical Academic Center of Trás-os-Montes and Alto Douro, University of Trás-os-Montes and Alto Douro, 5000-801 Vila Real, Portugal

**Keywords:** *Cannabis sativa*, colorectal cancer, cannabinoids, terpenes, apoptosis, proliferation, metastasis, inflammation, angiogenesis, oxidative stress, bioactive compounds

## Abstract

*Cannabis sativa* is a multipurpose plant that has been used in medicine for centuries. Recently, considerable research has focused on the bioactive compounds of this plant, particularly cannabinoids and terpenes. Among other properties, these compounds exhibit antitumor effects in several cancer types, including colorectal cancer (CRC). Cannabinoids show positive effects in the treatment of CRC by inducing apoptosis, proliferation, metastasis, inflammation, angiogenesis, oxidative stress, and autophagy. Terpenes, such as β-caryophyllene, limonene, and myrcene, have also been reported to have potential antitumor effects on CRC through the induction of apoptosis, the inhibition of cell proliferation, and angiogenesis. In addition, synergy effects between cannabinoids and terpenes are believed to be important factors in the treatment of CRC. This review focuses on the current knowledge about the potential of cannabinoids and terpenoids from *C. sativa* to serve as bioactive agents for the treatment of CRC while evidencing the need for further research to fully elucidate the mechanisms of action and the safety of these compounds.

## 1. Introduction

Cannabis is one of humanity’s oldest plants, yet it has also been a topic of discussion throughout history [1]. This plant is psychotropic and includes about 500 distinct chemical components, the most important of which are cannabinoids. The *Cannabis indica* Lam, *Cannabis ruderalis* Janisch, and *Cannabis sativa* Linnaeus are the species with the most psychotropic secondary metabolites. *C. ruderalis* yields smaller amounts of Δ^9^-tetrahydrocannabinol (Δ^9^-THC) and is less used in medicine, while *C. indica* is typically employed as a sedative, and *C. Sativa* as a psychoactive [2]. Among them, *C. sativa* is the most widely cultivated and exploited for a variety of purposes [3].

Cannabis for medicinal uses has recently been legalized in many countries. Plant material and extracts can be used to alleviate chronic pain and muscle spasms, reduce nausea during chemotherapy, improve appetite in HIV/AIDS patients, improve sleep, and reduce tics in Tourette’s syndrome patients. Moreover, it can be used in extreme cases of anorexia, arthritis, glaucoma, and inflammatory bowel disease [4]. The bulk of medicinal compounds is found in feminine inflorescences, and medicinal properties are commonly attributed to cannabinoids, although other bioactive substances, including terpenes, may also contribute to the health effects of cannabis [5]. According to the entourage effect theory, the medicinal benefits of cannabis are increased when all the plant’s constituents, such as terpenes, flavonoids, and cannabinoids, are present and interact with each other. Some researchers believe that the entourage effect may be especially important when it comes to the potential use of cannabis for cancer treatment [6]. In fact, cannabinoids and terpenes are known to have a whole range of potential health benefits, ranging from pain relief [7,8] to anti-inflammatory properties [9,10]. Thus, the high interest in these compounds is related to their numerous pharmacological properties. Regardless, this topic is far from being well understood.

Colorectal cancer (CRC) is one of the cancers with the highest incidence and mortality worldwide, due to the lack of early diagnosis methods and effective treatments. Cannabinoids are known to interact with the intestinal endocannabinoid system (ECS), which expresses CB1 and CB2 receptors, other G-protein-coupled receptors, endogenous ligands, and degrading enzymes [11]. It has been suggested that *C. sativa* can exert anticarcinogenic effects, through antiproliferative, anti-inflammatory, pro-apoptotic, antiangiogenic, and other mechanisms, including in CRC [12,13]. However, the research on the use of *C. sativa* bioactive compounds for the treatment of this cancer is still limited and inconclusive.

On the other hand, it is important to consider that cannabis consumption can also have adverse impacts on health, normally triggered by THC. These effects may include fatigue, tachycardia, nausea, dizziness, dry mouth, altered mood and behavior, psychomotor impairment, and visual and auditory hallucinations [14,15]. Furthermore, the long-term use of medicinal cannabis may increase the risk of substance use disorder and psychiatric comorbidities. It is also vital to remember that marijuana is still controlled in several countries where using it is prohibited [4].

This review summarizes the current knowledge regarding the potential benefits of cannabinoids, terpenes, and their mixtures from *C. sativa* for the prevention and treatment of CRC, focusing on their effects on apoptosis, proliferation, metastasis, inflammation, angiogenesis, oxidative stress, and autophagy. While the bioactivity of cannabinoids in CRC has been reviewed before [16,17,18], this work highlights the importance of understanding the effect of terpenes and their synergistic effects with cannabinoids to fully elucidate their mechanisms of action and safety.

## 2. Bioactive Compounds of *Cannabis sativa*

Among the multiple bioactive compounds found in *C. sativa*, the main ones are cannabinoids, terpenoids, flavonoids, stilbenoids, and alkaloids [19,20]. When consumed, these substances can induce a variety of beneficial health effects and are thought to contribute to the plant’s therapeutic qualities. As for natural products in general, the phytochemical content of *C. sativa* varies, depending on distinct factors, including genetics, growing conditions, stage of growth, harvest time, processing, and storage, among others [21]. *C. ruderalis* and *C. indica* contain a smaller amount of CBD than *C. sativa*. In contrast, *C. indica* has the largest THC content compared to *C. sativa*, and *C. ruderalis* has the lowest [2].

### 2.1. Cannabinoids

Cannabinoids are a type of terpenophenolic compounds with a C_21_ backbone [22]. The diversity of chemical structures of phytocannabinoids is mainly due to the differences between the isoprenyl groups, the side chain and the resorcinyl core [23]. Cannabinoids, according to their chemical structure, can be classified into 11 different classes: cannabigerol (CBG), cannabidiol (CBD), cannabichromene (CBC), Δ^9^-THC, Δ^8^-THC, cannabicyclol (CBL), cannabinol (CBN), cannabinodiol (CBND), cannabielsoin (CBE), cannabitriol (CBT), and miscellaneous-type cannabinoids [22,24].

**Figure 1:**
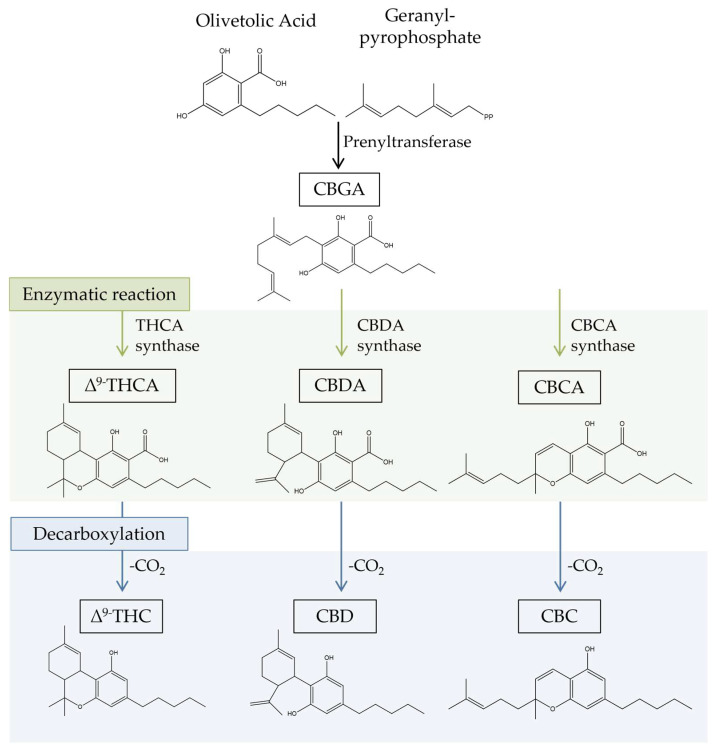
Biosynthesis of cannabinoids in *Cannabis*. CBC, cannabichromene; CBCA, cannabichromenic acid; CBD, cannabidiol; CBDA, cannabidiolic acid; CBGA, cannabigerolic acid; ∆^9^-THCA, ∆^9^-tetrahydrocannabinolic acid; ∆^9^-THC, Δ^9^-tetrahydrocannabinol.

Most cannabinoids originate from cannabigerolic acid (CBGA), as revealed in Figure 1. Its biosynthesis occurs with the production of CBGA from the alkylation of olivetolic acid with geranyl pyrophosphate (GPP). The primary cannabinoids, cannabichromenic acid (CBCA), cannabidiolic acid (CBDA), and ∆^9^-tetrahydrocannabinolic acid (∆^9^-THCA), are synthesized in acid form by the enzymes CBCA synthase, CBDA synthase, and THCA synthase. Decarboxylation then converts the acidic forms of cannabinoids into neutral forms (THC, CBD, and CBC), which are recognized for having higher pharmacological potential [2,25,26,27]. This conversion typically occurs naturally as the plant matures and dries, or because of heat exposure during processing or cooking [28].

The phytocannabinoids are essentially produced in glandular trichomes, mainly in female inflorescences. *C. sativa* mostly synthetizes cannabinoids of the alkyl type, characterized by a pentyl (C_5_) side chain and an isoprenyl (C_10_) monoterpene component. In general, Δ^9^-THC and CBD are the prevalent components of this plant and, based on the proportion of these two, three *C. sativa* chemotypes are known: (i) drug type, with predominant Δ^9^-THC; (ii) intermediate type, with similar amounts of both cannabinoids; and (iii) fiber type, with predominant CBD. CBGA, CBDA, and their decarboxylated derivatives are the main cannabinoids found in the fiber type [20].

Naturally, the quality and composition of *C. sativa* extracts vary among the *C. sativa* chemotypes. These are also highly dependent on other factors, including the extraction techniques employed. As an example, according to the study by Rožanc et al. [29], a methanolic soxhlet extraction, which requires higher temperatures than ultrasound-assisted extraction (UAE), supercritical fluid extraction CO_2_ (SFE-CO_2_), and ethanolic maceration, provided a higher quantity of decarboxylated cannabinoids in the final extract, i.e., in neutral form. In turn, the use of UAE, SFE-CO_2_, and maceration improved the levels of acid forms of cannabinoids in the extracts. In this sense, efforts must be made to optimize the extraction procedure, keeping in mind the desired target compositions [30].

Note that contrarily to Δ^9^-THC, CBD is psychotropically inactive and is of current interest due to its antiepileptic, antioxidant, and anti-inflammatory properties, being useful in neuropsychiatric disorders, and the attenuation of THC’s negative effects [31]. The main pharmacological effects of the major cannabinoids are summarized in Table 1.

### 2.2. Terpenes

**Figure 2:**
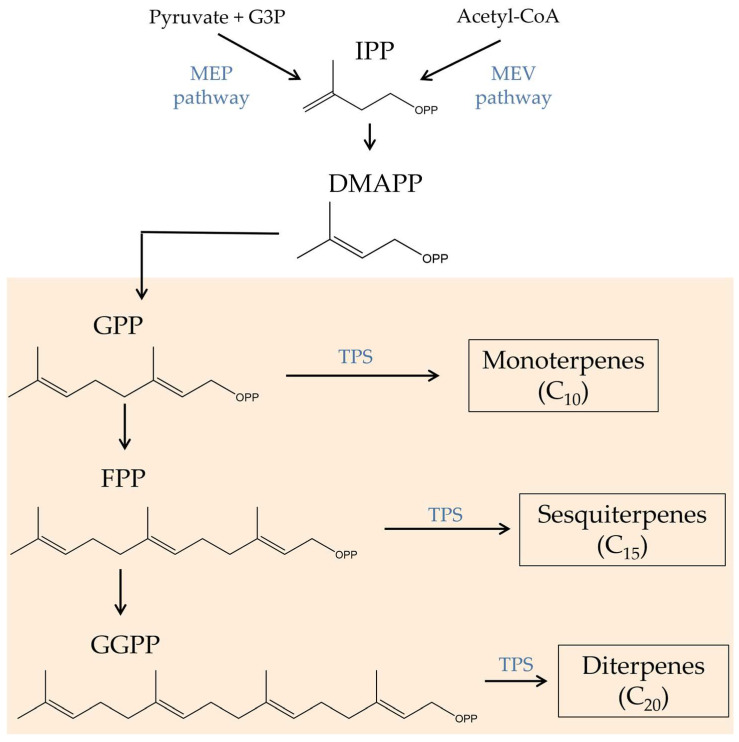
Biosynthetic route of terpenes. CBGA, cannabigerolic acid; DMAPP, dimethylallyl pyrophosphate; FPP, farnesyl pyrophosphate; G3P, glyceraldehyde 3-phosphate; GGPP, geranylgeranyl diphosphate; GPP, geranyl pyrophosphate; IPP, isopentenyl pyrophosphate; MEP, methylerythritol phosphate; MEV, mevalonic acid; TPC, terpene synthase.

Terpenes, characterized by multiple five-carbon isoprene units linked together to form a chain of hydrocarbons, comprise the second-largest class of plant constituents (120 identified so far). These secondary metabolites are synthesized via the isoprenoid biosynthesis metabolic pathway (Figure 2). Isopentenyl pyrophosphate (IPP) and its isomer dimethylallyl pyrophosphate (DMAPP), which are generated through the mevalonic acid (MEV) or methylerythritol phosphate (MEP) pathway, serve as the precursor molecules for this pathway. Afterward, a series of enzyme-catalyzed processes transform the IPP and DMAPP into several terpene precursors, including GPP, farnesyl pyrophosphate (FPP), and geranylgeranyl pyrophosphate (GGPP). GPP is responsible for the production of terpene molecules, which are then used in the production of cannabinoids. Different terpene synthases can further modify these precursors to produce a variety of monoterpenes, sesquiterpenes, and diterpenes [38,39].

**Figure 3:**
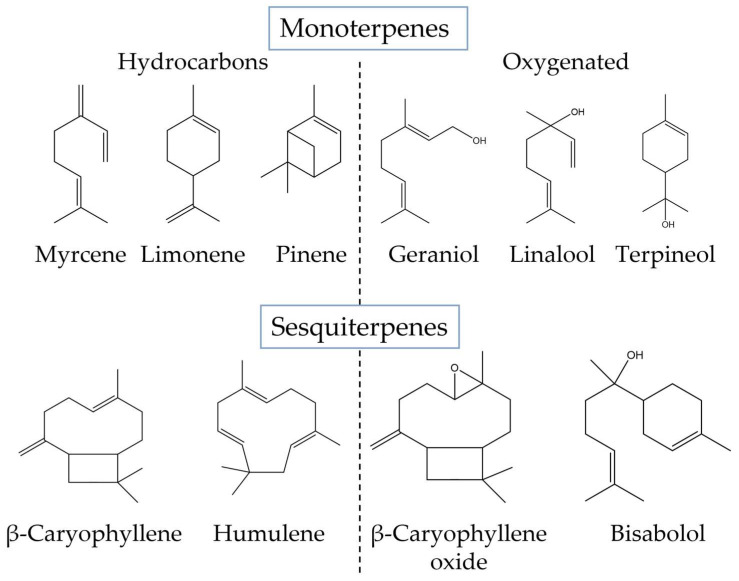
Chemical structure of the main terpenes found in *C. sativa*.

In general, these compounds are synthesized in different parts of the plant, such as the flowers, roots, leaves, and trichomes, with variable levels, which depend on abiotic and biotic factors [19]. Particularly in *C. sativa*, Chacon and coworkers found that terpenes may vary between 0.001 and 14.8 mg/g of the dry weight of the plant [39] and are in general rich in mono- and sesquiterpenes [34]. Monoterpenes, structurally characterized by a skeleton with two molecules of isoprene, are more volatile and contribute to the flavor and aroma of the plant, while sesquiterpenes (structure containing three molecules of isoprene) are more stable and contribute to the therapeutical benefits of the plant [40]. The monoterpene β-myrcene and the sesquiterpenes β-caryophyllene and α-humulene have been identified in most cannabis species, including *C. sativa*. Other typical terpenes synthesized by this species include α-pinene and limonene, among others (Figure 3) [41]. In addition, terpenoids (i.e., oxygenated terpenes) such as linalool, α-terpineol, guaiol, α- and β-bisabolol are often found in *C. sativa* [42]. Moreover, in a study by Janatová, among six genotypes of *C. sativa*, four were found to have pinocarveol in large amounts. Additionally, one of the genotypes contained significantly higher concentrations of the major terpenes (limonene, linalool, fenchol, and α-terpineol) when compared to the other genotypes. As a result, depending on the substances of interest, the genotype selected, as well as the type of extraction, should always be adjusted.

Studies have indicated that, in addition to cannabinoids, terpenes possess multiple biological potentialities such as antifungal, antiviral, anticancer, anti-inflammatory, antihyperglycemic, antiparasitic, antioxidant, and antimicrobial effects (Table 2).

## 3. Bioactive Compounds in the Prevention and Treatment of Colorectal Cancer

Studies on cancer prevention have typically focused on the inhibition of carcinogenic processes, such as the formation of aberrant crypt foci (ACF) [12] and CRC precursor lesions, as well as the inhibition of cancer cell proliferation [45]. On the other hand, studies on cancer therapy typically focus on inducing the apoptosis of cancer cells [46], inhibiting angiogenesis and metastasis [47], and reducing inflammation [48]. Cannabinoids and terpenes have been shown to be useful in both the chemoprevention and the treatment of CRC, making them an attractive therapeutic option.

### 3.1. Effects of Cannabinoids in CRC-Associated Mechanisms

**Figure 4:**
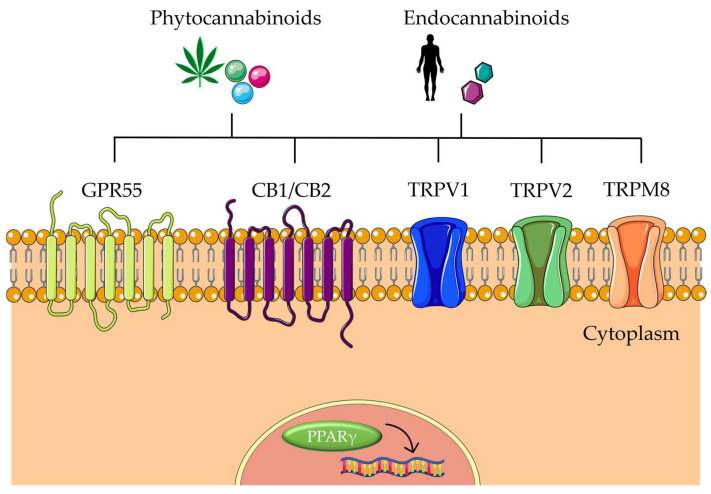
Schematic representation of endocannabinoid system components. GPR55, G-protein-coupled receptor 55; PPARs, peroxisome proliferator-activated receptor; TRPM8, transient receptor potential cation channel subfamily M member 8; TRPV1, transient receptor potential cation channel subfamily V member 1. The figure was partly generated using Servier Medical Art, provided by Servier, licensed under a Creative Commons Attribution 3.0 unported license.

Over history, there has been increasing evidence of cannabis’s beneficial effects on CRC. Several in vitro and in vivo experiments showed that natural phytocannabinoids from *C. sativa* can interact with some of the mechanisms inherent in cancer. Among them, apoptosis, autophagy, inflammation, migration, invasion, and metastasis are the target mechanisms that have demonstrated better outcomes in CRC carcinogenesis with cannabinoids (Table 3). As mentioned earlier, cannabinoids can exert anticancer effects in part due to their interaction with the ECS. This is a complex cell signaling system present in all mammals, which is involved in regulating various physiological and cognitive processes, such as appetite, pain, mood, and memory. It is composed of three main components: endocannabinoids, receptors, and metabolic enzymes. Endocannabinoids are lipid-based neurotransmitters produced by the body, such as anandamide and 2-arachidonoylglycerol (2-AG), which bind to cannabinoid receptors (CB1 and CB2) found on the surface of cells, triggering a cellular response. Cannabinoids then can act on the ECS by mimicking endocannabinoids and binding to CB1 and CB2 receptors. Other receptors interact with endocannabinoids and modulate the ECS, including G-protein-coupled receptor 55 (GPR55), transient receptor potential cation channel subfamily V member 1 (TRPV1), TRPV2, transient receptor potential cation channel subfamily M member 8 (TRPM8), and peroxisome proliferator-activated receptors (PPARs) (Figure 4) [49]. The role of ECS in CRC has been reviewed elsewhere [17]. However, it is important to note that CRC cells and tissues express both CB1 and CB2 receptors, and TRPM8, TRPA1, TRPV1, and TRPV2 receptors, to which cannabinoids can bind to exert biological effects on CRC [12,17].

#### 3.1.1. CBD

Isolated CBD is the most extensively studied cannabinoid in cancer research due to its numerous health benefits and non-psychoactive nature. This cannabinoid was described as cytotoxic in different types of CRC cells, inhibiting their viability, namely in HCT116, SW480, SW620, CACO-2, HT-29, and DLD-1 [46,50,51,52]. Lee et al. found that CBD suppressed cell viability through a mechanism dependent on the CB2 receptor and not on CB1. Additionally, normal human colon cells resisted CBD, establishing its safety for noncancer cells [50]. Furthermore, several CRC cell lines (CACO-2, HT-29, DLD-1, SW620, SW480, COLO205, and HCT116) suffered significant reductions in proliferation after treatment with CBD at different concentrations (2.5–15 µM) [47,50,52,53,54,55]. In this way, its beneficial effects have become a target of study in different anticancer mechanisms.

**Figure 5:**
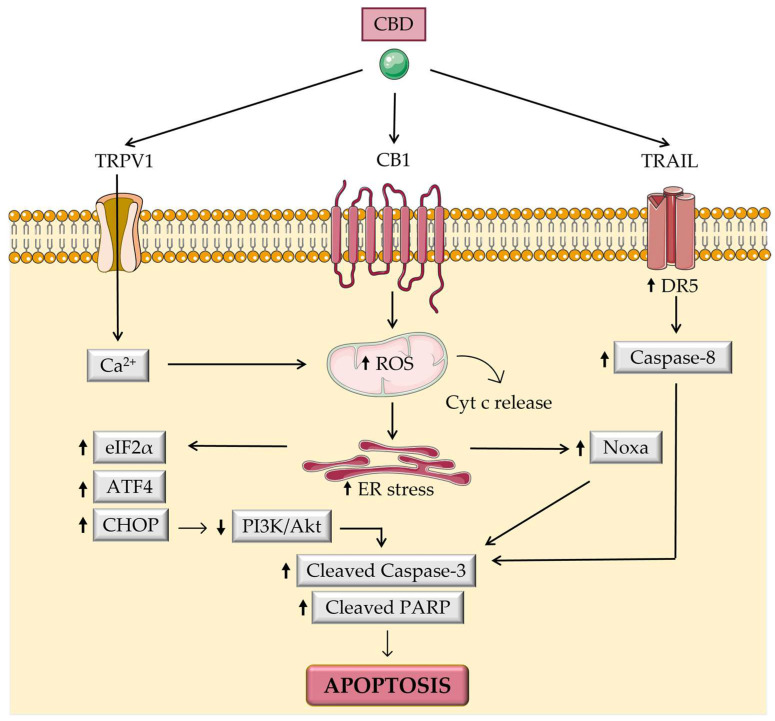
Effect of CBD on pro-apoptotic mechanisms in colorectal cancer. ⬆, increase; ⬇, decrease; ATF4, activating transcription factor 4; CHOP, C/EBP homologous protein; eIF2α, phosphorylated eukaryotic initiation factor 2α; PARP, poly (ADP-ribose) polymerase; PI3K/Akt, phosphoinositide 3-kinase/protein kinase B; ROS, reactive oxygen species; TRPV1, transient receptor potential cation channel subfamily V member 1. The figure was partly generated using Servier Medical Art, provided by Servier, licensed under a Creative Commons Attribution 3.0 unported license.

Resistance to apoptosis (cell death) is a hallmark of all types of cancer, so several apoptosis-inducing drugs have been developed as cancer therapies, including for CRC [56]. Aviello et al. [54] corroborated that CACO-2 CRC cells, treated with CBD (10 μM for 24 h), significantly decreased the expression of phospho-protein kinase B (Akt) (*p* < 0.001) and upregulated caspase-3 expression in colonic tissues of azoxymethane (AOM)-induced mice (CBD, 1 mg/kg). These data suggest that CBD can induce apoptosis involving the phosphoinositide 3-kinase (PI3K)/Akt signaling pathway, essential in the regulation of cell growth, migration, differentiation, and apoptosis. In other CRC cell lines, namely, HCT-116, SW480, and SW620, this mechanism occurred through the antagonism of several receptors, including CB1, TRPV1, and PPARγ [50]. This study conducted by Lee and coworkers revealed that CBD arrested the cell cycle by lowering levels of cyclin-dependent kinases (CDKs) and cyclins, specifically cyclin D1 and D3, CDK2, CDK4, and CDK6, showing antiproliferative and pro-apoptotic properties. Additionally, CBD increased caspase-3/7 activity in a dose-dependent manner (5 and 10 µM). In the same study, authors also revealed that induced apoptosis was also mediated by the increased expression of endoplasmic reticulum (ER) stress proteins, including binding immunoglobulin protein, inositol-requiring enzyme 1α, phosphorylated eukaryotic initiation factor 2α, activating transcription factor 3 (ATF3), and ATF4. A similar trend was found in a study realized by Jeong et al. in HCT116 and DLD-1 cells [46]. In fact, they demonstrated that increased reactive oxygen species (ROS) production contributed to an increment in Noxa levels via ER stress. Thus, the authors concluded that the induced apoptosis was mediated by Noxa activation, which led to caspase activation and poly (ADP-ribose) polymerase (PARP) cleavage. Consistent with that, Noxa levels were also elevated in the tumor tissues of female BALB/c nude mice (xenograft: HCT116), validating the Noxa-mediated apoptosis in an in vivo model. Moreover, Kim et al. [51] reported that CBD increased apoptosis induced by tumor necrosis factor (TNF)-related apoptosis-inducing ligand (TRAIL), increasing the expression of death receptor 5 through ER stress in HCT116, HT29, and DLD-1 cells. In vivo results also confirmed the rise in DR5 and C/EBP homologous protein (CHOP) expression, an ER stress-related protein, in tumor tissues. Additionally, it was shown that the combination of CBD and TRAIL significantly reduced the cells’ ability to form colonies compared to controls (*p* < 0.001). Sreevalsan et al. [53] also proved that phosphatases may also be induced or activated by CBD, namely dual-specificity protein phosphatase 10 (DUSP10), prostatic acid phosphatase (ACPP), and protein tyrosine phosphatase non-receptor type 6 in SW480 cells at 15 µM. In summary, the previous results suggest that CBD-induced apoptosis can occur through a variety of mechanisms, including the inhibition of the Akt and extracellular signal-regulated kinase (ERK) signaling pathways, which are important for cancer cell survival, as well as the activation of caspase-3, an enzyme involved in the apoptosis process (Figure 5).

It is well established that oxidative stress and the generation of ROS are significant factors in apoptosis, and this cellular process can be delayed or prevented by some antioxidants [57]. A study confirmed that CBD caused oxidative stress in HT-29 cells, probably through the generation of ROS, which depleted glutathione (GSH) and inhibited the activities of catalase, glutathione reductase (GR), and glutathione peroxidase (GPx). These findings were supported by the observation that malondialdehyde (MDA) levels were markedly increased (*p* < 0.01) in cells treated with CBD (2.6 ± 0.18 nmol/mL) compared to control cells (1.6 ± 0.27 nmol/mL), while they were largely unaltered in HT-29 cells exposed to THC [52]. Combined with oxaliplatin, a chemotherapeutic drug for cancer, CBD (4 μM CBD for 24 h) also produced similar results in oxaliplatin-resistant DLD-1 and colo205 cells [55]. In contrast, an in vivo study showed that CBD induced a significant increase in the activities of superoxide dismutase, GPx, and GR, and simultaneously decreased MDA levels (*p* < 0.05) in BALB/c mice (xenograft model, CT26). These events may be linked to other CRC protective mechanisms against inflammation. Indeed, interleukin-6 (IL-6) and IL-8 levels, i.e., pro-inflammatory cytokines, were noticeably lower in the 5 mg/kg CBD-treated groups compared with the CRC groups (*p* < 0.05) [58]. The anti-inflammatory potential of CBD has already been confirmed in several models of colitis [17]. However, a study carried out by Nallathambi et al. [59] revealed that treatment with purified CBD, at different concentrations (16–252 µg/mL), resulted in a significant decrease in IL-8 levels at lower concentrations of CBD, while no anti-inflammatory activity was found for the higher CBD concentrations in HCT116 cells. Hence, more research must be undertaken to better understand the anti-inflammatory effects of CBD on CRC.

Autophagy and apoptosis are linked and can interact with each other. Autophagy can prevent or delay apoptosis, and apoptosis can also trigger autophagy. Both processes help regulate cell survival and death, contributing to cellular homeostasis maintenance [60]. CBD has also been shown to induce autophagy in oxaliplatin-resistant DLD-1, colo205 cells, and tumor tissues of BALB/c nude mice (xenograft model, colo205) [55]. Microtubule-associated proteins light chain 3 (LC3) and p62 expression, which are commonly used autophagic biomarkers, were both considerably enhanced by the combination of oxaliplatin and CBD in CRC cells. Additionally, the proportion of autophagic cells significantly increased (*p* < 0.001). In vivo results also revealed a significant increase in microtubule-associated protein LC3 levels in colorectal tumors in oxaliplatin- and CBD-treated mice than in tumors in control mice. This experiment also proved that the cell’s response to oxaliplatin was increased when combined with CBD treatment, suggesting that this cannabinoid can be applied in combination with chemotherapeutic drugs in CRC to increase pharmacological effects.

Angiogenesis, the recruitment of new blood vessels, is a therapeutic target and an integral component of tumor development, invasion, and metastasis. This process engages a variety of growth factors, including integrins, chemokines, vascular endothelial growth factor (VEGF), and fibroblast growth factor [61]. Honarmand et al. reported that CBD treatments (1 and 5 mg/kg) decreased VEGF expression (*p* < 0.05) in a dose-dependent manner in tumor tissues of CRC-induced mice when compared to cancer control groups [58]. Regardless, few studies have been carried out regarding CBD and angiogenesis; thus, more research is needed to understand the effects of CBD on angiogenesis, an anticancer mechanism.

As stated earlier, adhesion, migration, and invasion mechanisms are involved in metastasis and the spread of cancer to other tissues. In HCT116 CRC cells, CBD at 1 (*p* < 0.05) and 2.5 µM (*p* < 0.001) caused a significant reduction in GPR55-dependent adhesion and migration to endothelial cells, suggesting that CBD’s antagonistic activity in GPR55 plays a key role in the reduction in metastasis [62]. In addition, Feng and coworkers reported that CBD could prevent the epithelial–mesenchymal transition in HCT116 cells, by potentiating E-cadherin and inhibiting *N*-cadherin, snail, vimentin, and hypoxia-inducible factor 1-α. In HCT116, SW620, and DLD-1 CRC cells, the expression of adenomatous polyposis coli and casein kinase 1 was likewise suppressed, while Axin1 was increased. Overall, these data indicate that CBD has anti-invasion and antimetastatic potential through the Wnt/β-catenin signaling pathway [47].

All the aforementioned mechanisms could be responsible for the potential of CBD treatment to slow the growth of CRC tumors. Indeed, several CRC xenograft animal models (DLD-1, HCT116 and CT26), after treatment with CBD (1–20 mg/kg body weight), experienced a delay in tumor growth, as well as in volume and weight [46,47,51,58]. Furthermore, treatment with CBD (1 and 5 mg/kg) suppressed the development of aberrant crypt foci, CRC precursor lesions, and polyps in AOM-induced CRC mice [54].

It was reported that the administration of CBD can have some side effects, including the inhibition of hepatic drug absorption, changes in vitro cell viability, and decreases in fertilization capability. Despite these potential side effects, research has indicated that controlled administration of CBD in humans and animals is generally safe [63]. Therefore, more studies should be conducted for a better understanding of the possible side effects of CBD administration in the treatment of CRC.

#### 3.1.2. Δ^9^-THC

THC has also been reported to have good outcomes in inhibiting cell viability on CRC cells, namely, SW480, HCT-15, HT-29, and HCA7 [52,64]. In HT-29 cells, THC suppressed cell viability more than CB83 (a synthetic cannabinoid) after 24 h of treatment at 30 µM [52]. Additionally, Shor and coworkers reported that Δ^9^-THC and Δ^8^-THC were more toxic to polyp-derived cells than other cannabinoids, namely, CBD, CBC, CBDV, THCA, CBG, and other minor cannabinoids [65]. Furthermore, THC at 30 µM was shown to trigger significant inhibition of proliferation at 24 h (70.9 ± 5.59, *p* < 0.01) in HT-29 CRC cells, compared to untreated cells [52].

The induction of apoptosis by THC through the activation of the CB1 receptor in CRC cells was first reported in 2007 by Greenhoug et al. [64]. The authors suggested that apoptosis was induced by the activation of the B-cell lymphoma-2 (Bcl-2) family member BAD protein dependent on the CB1-mediated Ras-mitogen-activated protein kinase and PI3K/Akt pathway inhibition. In reality, the inhibition of ERK and Akt activity by THC was complemented by activation of the proapoptotic BAD. Additionally, THC increased the simultaneous cleavage of the caspase-3 substrate PARP. Through staining with 4′,6-diamidino-2-phenylindole, morphological changes such as chromatin condensation and micronucleation were seen in cells treated with THC. Cerretani et al. [52] supported the evidence that THC acts through the CB1 receptor in inducing cytotoxicity in HT29 cells.

In addition to the previous effects, THC was also claimed to exhibit anti-inflammatory and antitumoral potential in AOM/dextran sodium sulfate (DSS)-induced female C57BL/6 mice [48]. After treatment with THC (10 mg/kg body weight), the animals’ colons had a significant reduction in the severity of inflammation and tumor induction, as indicated by hematoxylin and eosin staining. Furthermore, this cannabinoid caused a reduction in IL-22 produced mainly by intra-epithelial cells, a cytokine that plays an important role in the severity of inflammation-induced colon cancer.

Optimizing the delivery method can improve the efficacy of THC treatment for CRC owing to its lipophilicity, tar-like viscosity, and instability. In fact, De la Ossa et al. suggested an alternative method of THC delivery. The oil-in-water emulsion solvent evaporation method used by the authors to encapsulate THC into biodegradable microspheres revealed its ability to prevent cancer cell proliferation in CACO-2 cells [66].

Due to THC’s psychoactive effects, clinical research has linked certain negative effects with its use, including dysphoria, depersonalization, anxiety, panic attacks, and paranoia [67]. Long-term use and high doses have been associated with most of these negative consequences. According to studies on cannabinoid effects, some of the harmful effects of THC may be mitigated by CBD. However, the results are not always consistent [68]. More research is needed to determine if THC can be used in combination with other treatments, such as chemotherapy, to improve outcomes. Studies should also evaluate the potential long-term effects of THC use in patients and/or animals with CRC.

#### 3.1.3. CBG

CBG, a non-psychoactive cannabinoid, has gained a lot of interest due to its ability to be a partial agonist of CB1 and CB2 receptors [69]. In addition to the main receptors of the endocannabinoid system, CBG also acts as an antagonist at TRPM8, TRPV4, and 5HT_1A_ and an agonist at TRPA1, TRPV1, and TRPV2 receptors [70].

In CRC, CBG treatment had positive results in cell survival, apoptosis, oxidative stress, and in vivo tumor growth. As for cell survival, CBG was reported to induce a significant decrease in the viability of CACO-2 cells at 30 µM after 3 h of incubation (*p* < 0.001). However, after 48 h of incubation, CBG effects on cell viability were significantly decreased even for lower concentrations (3, 10, and 30 µM). In HCT116 cells, a similar trend was observed, and this effect was repressed in cells where TRPM8 was suppressed [12]. In HCT116 cells, purified CBG exhibited antiproliferative potential at concentrations 2.5, 5, and 10 µM [42]. Furthermore, an increase in CHOP mRNA and caspase-3 activity validated the apoptosis-inducing effects of CBG on CACO-2 cells. Additionally, Borrelli et al. [12] demonstrated that CBG could increase the production of ROS in CRC cells, reduce tumor growth in a xenograft model, and prevent abnormal crypt foci from developing in an AOM-induced model.

Despite the small number of studies evaluating CBG’s effects on CRC, there is some evidence suggesting its potential as a chemopreventive agent. Its potential for the treatment of breast cancer and melanoma has also been highlighted [33], but additional research is required to elucidate its anticancer mechanisms and its potential in CRC.

#### 3.1.4. Minor Cannabinoids

Despite being less studied, minor cannabinoids such as CBDV, CBL, CBGV, CBCA, THCV, and CBGA revealed positive effects in the cell survival and proliferation of CRC cells. Ben-Ami Shor et al.’s research [65] indicated that the combinations of CBCA (14.5 or 29 μM), CBDV (23.5 or 47 μM), THCV (20 or 40 μM), and CBGA (25.6 or 51.2 μM) triggered a decrease in the cell viability of cells derived from human polyps. The combination of CBCA and CBDV also had synergistic effects, as well as the combination of THCV and CBGA. In HCT116 cells, data revealed cell viability proportions of 93, 87, and 51% with CBDV; 69, 44, and 45% with CBL; and 93, 75, and 71% for CBGV treatments at 2.5, 5, and 10 μM, suggesting that these cannabinoids exerted antiproliferative effects against CRC cells [50]. Despite the antiproliferative properties demonstrated for the cannabinoids CBCA, CBDV, THCV, and CBGA, the concentrations were relatively high compared to those of CBD, THC, and CBG, suggesting that their effects are not very strong. Moreover, studies have confirmed that these cannabinoids possess a variety of anti-inflammatory and anticancer properties [32,33,34], but again, their benefits on CRC were not significant, which emphasizes the importance of further researching and understanding the dose-related effects of minor bioactive compounds from *C. sativa*.

**Table 3:** Anti-CRC effects of isolated cannabinoid-rich *C. sativa* naturally occurring phytocannabinoids.

Cannabinoids	Cell Line/Animal Model	Doses	Cancer-Associated Mechanisms	Effects on CRC	Refs.
CBD	HCT-116, SW480, and SW620	0–10 μM	Cell survival Proliferation Apoptosis	⬇ cell viability, proliferation (2.5, 5, 10 μM)**,** cyclin D1/D3, CDK2, CDK4, CDK6; ⬆ G1 phase arrest, p-eIF2α, ATF3, ATF4, cleaved caspase-3/7 and PARP	[50]
	CACO-2, HT-29, and DLD-1 BALB/c nude mice (Xen: DLD-1)	In vitro: 4 μM In vivo: 10 mg/kg	Cell survival Proliferation Apoptosis Tumor growth	In vitro: ⬇ cell viability, TRAIL-induced colony formation; ⬆ apoptosis, DR5, PERK, eIF2α, ROS, CHOP, cleaved caspase-3, caspase-8 and PARP In vivo: ⬇ tumor growth, ⬆ apoptosis, DR5, CHOP	[51]
	CACO-2, HT-29, HTC116	16–252 μg/mL	Apoptosis Inflammation	⬇ IL-8; ⬆ apoptosis	[59]
	CACO-2 and HCT116 AOM-induced ICR mice	In vitro: 0.01–10 μM In vivo: 1 and 5 mg/kg	Proliferation Apoptosis Tumor growth Genotoxicity	In vitro: ⬇ proliferation; H_2_O_2_-induced DNA damage, phospho-Akt; ⬆ caspase-3 In vivo: ⬇ ACF, polyps, tumor formation, Akt phosphorylation	[54]
	HCT116, SW620, and DLD-1 BALB/c nude mice (Xen: HCT116)	In vitro: 3,6, and 12 μM In vivo: 10 and 15 mg/Kg	Proliferation Metastasis Tumor growth	In vitro: ⬇ proliferation, migrated cells, invasive cells, N-cadherin, vimentin, Snail, β-catenin, APC, CK1; ⬆ E-cadherin, β-catenin, Axin1 In vivo: ⬇ tumor volume, weight, vacuole degradation, edge collection	[47]
	HT-29	0.1 mM–0.1 nM	Cell survival Proliferation Apoptosis Oxidative stress	⬇ cell viability, proliferation, GR, GPx, CAT, GSH/GSSG ratio; ⬆ MDA, necrotic cells	[52]
	HCT116 and DLD-1 Female BALB/c nude mice (Xen: HCT116)	In vitro: 0–8 μM In vivo: 10–20 mg/kg	Cell survival Proliferation Apoptosis Oxidative stress Tumor growth	In vitro: ⬇ cell viability, colony formation, SOD, CAT; ⬆ apoptosis (6 μM), Noxa levels (6 μM), p53, ROS, superoxide, IRE1α, PERK, BiP, GRP94, CHOP, cleaved caspase-3/8/9 and PARP; In vivo: ⬇ tumor growth; ⬆ Noxa levels	[46]
	Male BALB/c mice (Xen: CT26)	1 and 5 mg/kg	Inflammation Oxidative stress Angiogenesis Tumor growth	Tumor tissue: ⬇ tumor growth, cellular pleomorphism, VEGF; ⬆ apoptosis (5 mg/kg) Blood: ⬇ IL-6, IL-8 (5 mg/kg), MDA; ⬆ SOD, GPx, GR, TAC	[58]
	SW480	15 μM	Proliferation Apoptosis	⬇ proliferation; ⬆ mRNA expression of *DUSP1*, *DUSP10*, *ACPP*, *ACPP, PTPN6,* cleaved caspase-3 and PARP	[53]
	Oxaliplatin-resistant DLD-1 and colo205 BALB/c nude mice (Xen: colo205)	In vitro: 0–30 μM In vivo: 10 mg/kg	Proliferation Apoptosis Oxidative stress Autophagy Mitochondrial dysfunction	In vitro: ⬇ proliferation, AKT, TOR, AMPK, NOS3, NO, SOD; ⬆ cell death, LC3, p62, rapamycin, autophagic cells, ROS, mitochondrial dysfunction In vivo: ⬇ tumor size, SOD, phopho-NOS3; ⬆ LC3	[55]
	HCT116	1–2.5 μM	Metastasis	⬇ GPR55-dependent adhesion and migration	[62]
THC	SW480, HCT-15, HT-29, HCA7	2.5–12.5 μM	Cell survival Apoptosis	⬇ cell viability, ERK, AKT; ⬆ chromatin condensation, micronucleation, BAD dephosphorylation, cleaved caspase-3 and PARP	[64]
	HT-29	0.1 Mm–0.1 nM	Cell survival Proliferation Apoptosis Oxidative stress	⬇ cell viability, proliferation, CAT; ⬆ necrotic cells, GR, GPx	[52]
	AOM/DSS-induced female C57BL/6 mice	10 mg/kg	Inflammation Tumor appearance	⬇ inflammation severity, IL-22, no tumors on treated mice	[48]
CBG	CACO-2 and HCT 116 ICR mice (Xen: HCT116 and AOM-induced)	1–30 μM	Cell survival Apoptosis Oxidative stress Tumor growth	In vitro: ⬇ cell viability (3, 10, 30 μM); ⬆ apoptosis, CHOP mRNA, ROS In vivo: ⬇ tumor growth	[12]
	HCT116	0–10 μM	Proliferation	⬇ proliferation (2.5, 5, 10 μM)	[50]
CBDV, CBL, CBGV	HCT116	0–10 μM	Proliferation	⬇ proliferation (2.5, 5, 10 μM)	[50]
CBCA, CBDV, THCV, CBGA	Polyp-derived cells	14.5–51.2 μM	Cell survival	⬇ cell viability in combined CBCA (14.5, 29 μM), CBDV (23.5, 47 μM), THCV (20, 40 μM), CBGA (25.6, 51.2 μM)	[65]

⬆, increase; ⬇, decrease; ACF, aberrant crypt foci; AMPK, 5’ AMP-activated protein kinase; AOM, azoxymethane; APC, adenomatous polyposis coli; Akt, protein kinase B; ATF, activating transcription factor; BAD, Bcl-2 associated agonist of cell death; Bcl, B-cell lymphoma 2; BiP, immunoglobulin protein; CAT, catalase; CBD, cannabidiol; CBDA, cannabidiolic acid; CBDV, cannabidivarin; CBG, cannabigerol; CBL, cannabicyclol; CBGV, cannabigerovarin; CBN, cannabinol; CDK4, cyclin-dependent kinase 4; CHOP, C/EBP homologous protein; CK1, casein kinase 1; CO_2_, carbon dioxide; DSS, dextran-sodium sulphate; DR5, death receptor 5; eIF2α, eukaryotic initiation factor 2; EtOH, ethanol; GPx, glutathione peroxidase; GR, glutathione reductase; GSH, reduced glutathione; GSSG, oxidized glutathione; IL-8, interleukin-8; IRE1α, inositol-requiring enzyme 1α; LC3, microtubule-associated protein 1A/1B-light chain 3; MDA, malondialdehyde; NOS3, nitric oxide synthase 3; PARP, poly (ADP-ribose) polymerase; PERK, protein kinase RNA-like endoplasmic reticulum kinase; ROS, reactive oxygen species; SOD, superoxide dismutase; TAC, total antioxidant capacity; Δ^9^-THC, Δ^9^-tetrahydrocannabinol; TOR, target of rapamycin; TRAIL, TNF-related apoptosis-inducing ligand; TNF-α, tumor necrosis factor α; VEGF, vascular endothelial growth factor.

### 3.2. Effects of Terpenes on CRC

Terpenes have been screened for their potential to prevent CRC when used alone or, in the case of the *C. sativa* plant, to enhance the plant’s medicinal properties. These compounds act by inhibiting the growth and proliferation of cancer cells, and by inducing apoptosis. In addition, there is evidence that some terpenoids may have similar effects to cannabinoids, such as THC, in terms of their ability to interact with the endocannabinoid system. This interaction can cause changes in the activity of certain receptors and enzymes, which in turn affect a wide range of physiological processes. Indeed, β-caryophyllene was shown to interact with the CB2 receptor, acting as its full agonist, resulting in a reduction in pain and inflammation in male rats and mice [71]. Furthermore, in a study conducted by LaVigne et al., the terpenes α-humulene, β-pinene, and β-caryophyllene increased the bioactivity of cannabinoids through interaction with the CB1 receptor [72].

One of the most widely studied terpenes in the context of CRC is β-caryophyllene (BCP). A study performed by Dahham et al. [73] determined that BCP displayed antiproliferative and pro-apoptotic effects on colon cancer cells (HCT116). Treatment with BCP at 20 μM for 24 h induced a significant suppression of the expression of antiapoptotic proteins, including survivin and X-linked inhibitor of apoptosis protein (XIAP), the serine protease high-temperature requirement factor and a member of heat shock proteins family, HSP60 (*p* < 0.001). In addition, BCP treatment, at the dose of 200 mg/kg, remarkably reduced the tumor size (0.07%) in an in vivo xenograft model (HCT116 cells). In parallel, the histological analysis of the tumors from the BCP-treated mice revealed a clear reduction in the thickness of the blood vessels compared to untreated tumors. Moreover, BCP was also reported to suppress the activation of signaling pathways involved in cancer development and progression, such as the Akt pathway. A study performed by Zhou et al. [74] revealed that BCP (at 50 μM for 48 h) reduced the levels of p-Akt, p-mTOR, protein 3-phosphoinositide-dependent protein kinase-1, and lactate dehydrogenase A in CT26 cells under high-glucose conditions (*p* < 0.05).

Inflammatory bowel diseases are closely associated with an increased risk of CRC [75]. In this sense, Bento et al. [76] emphasized that the preventive treatment of DSS-induced colitis CD1 mice with BCP (50 mg/kg) resulted in a significant decrease in TNF-α, IL-1β, keratinocyte-derived chemokine, and interferon-γ protein levels (*p* < 0.05) in mice colon segments. These proteins are mediators involved in cellular migration and adhesion. The authors also revealed that the decrease in the inflammatory mediators was associated with the factor nuclear kappa B (NFκB) signaling pathway, as BCP decreased the activation of the p65 NFκB subunit. However, according to the findings above related to CBD and THC, further research should be conducted in cell lines and animal models of CRC to better understand the anti-inflammatory action of BCP in CRC. Indeed, DSS only induces colitis. In animal models of CRC associated with inflammation, DSS is administered with AOM [77].

Furthermore, the combination of terpenes and other drugs was found to provide a synergistic effect in reducing tumor growth in CRC. In this regard, Legault et al. [78] found that the combination of BCP (2.5 and 10 mg/mL) and paclitaxel (0.025 mg/mL) was more effective in inhibiting the growth (17.3 ± 0.2%) of CRC DLD-1 cells than the drug applied individually. BCP also increased the cytotoxicity of α-humulene in breast cancer MCF-7 cells. α-humulene (32 mg/mL) inhibited cell growth by 50 ± 6% alone, and by 75 ± 6% when combined with 10 mg/mL of BCP. Additionally, β-caryophyllene-oxide and α-humulene were shown to improve the antiproliferative effects of 5-fluorouracil and oxaliplatin in CACO-2 and SW620 cell lines [79].

D-limonene is another terpene found in cannabis that has been claimed to exhibit antitumor effects in CRC. In particular, in a study by Jia and coworkers, the authors described that D-limonene caused a reduction in the viability of colon cancer cells (LS174T) in a dose-dependent manner (0.4–3.2 mmol/L) [80], through apoptosis induction. This event was mediated through the activation of caspase-3, -9, and PARP cleavage, the increase in Bcl-2-like protein 4 (Bax) protein and cytosol cytochrome c from mitochondria, the decrease in Bcl-2 protein, and the suppression of the PI3K/Akt pathway. Additionally, Kawamori et al. [81] verified that the treatment of AOM-induced F344 rats with D-limonene, at 0.5%, significantly reduced (*p* < 0.001) the number of ACF when compared to cancer animals (157.2 ± 28.2 vs. 234.6 ± 63.2, respectively), a type of precancerous lesions, in the colon of rats administrated with AOM, suggesting that D-limonene may have chemopreventive potential in CRC.

Although less studied, there is some evidence that other terpenes found in *C. sativa* may hold the potential for fighting CRC. In this regard, myrcene was reported to be cytotoxic against colon cancer cells (HT-29) at concentrations higher than 200 μg/mL [82]. α-pinene also proved to inhibit tumor growth in Balb/c mice allografted with colon cancer CT-26 cells via NK cell activation [83]. Geraniol, in addition to increasing the cytotoxicity of 5-fluorouracil treatment in CACO-2 cells (geraniol, 400 µM and 5-fluorouracil, 5 µM) [84], reduced 53% of the tumor volume in Swiss nu/nu female mice with TC118 colorectal tumor grafts obtained from patients with CRC (geraniol, 150 mg/kg and 5-fluorouracil, 20 mg/kg) [85]. Linalool was found to significantly reduce the growth, migration, and invasion of colon cancer cells (HCT116 and SW480) and increase cell death, an effect probably mediated by the Akt/mTOR and Janus kinase 2 (JAK2)/signal transducer and activator of transcription 3 signaling pathways [86].

Compared with the effects of cannabinoids, several results were similar in different CRC cell lines and animal models. The antiproliferative and pro-apoptotic effects of BCP on HCT116 cells were also observed for CBD, CBG, CBDV, CBL, and CBGV. The decrease in cell growth in HT29 was induced by myrcene, CBD, and THC. In addition, limonene led to a decrease in ACF, as did CBD in a chemically induced AOM animal model. However, linalool, in addition to the anticancer mechanisms induced by cannabinoids in the SW480 cell line, also led to a reduction in cancer cell invasion and migration. Additionally, D-limonene reduced the viability of colon cancer cells (LS174T), not demonstrated for cannabinoids.

Despite studies claiming promising potential options for the treatment of CRC, it is necessary to note that most of the studies to date have been conducted in vitro, and more research, including in vivo studies, is needed to fully understand the potential benefits of terpenes in the treatment of CRC alone or combined with other drugs. Furthermore, the interactions between terpenes and cannabinoids should be further studied for a better understanding of their effects on CRC, i.e., whether their interaction enhances or counteracts the effects of the plant on this type of cancer.

### 3.3. Effects of Cannabis sativa Extracts on CRC

Interactions between terpenes/terpenoids and cannabinoids may intensify their pharmacological effects. In reality, there is growing evidence that these substances interact more favorably when used in combination than when used alone. This is known as the “entourage effect” in modern parlance, an effect attributed to cannabis’s medical properties [43]. Although little explored, several *C. sativa* extracts rich in cannabinoids and terpenes have been claimed to exert positive effects to fight CRC (Table 4).

Janatová et al. [42] suggested that the compounds responsible for the cytotoxicity of *C. sativa* ethanolic (EtOH) extracts in CACO-2 and HT29 cell lines were THC and CBD. However, in the same work, a specific genotype containing high concentrations of myrcene, β-elemene, β-selinene, and α-bisabolol oxide positively affected the selectivity of cytotoxic activity, due to synergistic effects. In another study conducted by Nallathambi and coworkers, the authors reported an increment in cytotoxic effects of a *C. sativa* EtOH extract in CRC cells (HCT 116, HT-29, and CACO-2) when this was combined with a THCA-rich fraction [45]. In addition to the EtOH extract, fractions obtained from it were also evaluated. The combination of a CBGA-rich fraction and a THCA-rich fraction resulted in a noticeable increase in apoptosis. The CBGA-rich fraction included other compounds such as CBN (3.67%), CBCA (3.53%), terpenes (0.72%), diterpenes (0.33%), and short free fatty acids (0.37%), which may have played a role in the outcome. Nallathambi et al. [59] conducted another study suggesting that a THCA-rich fraction and an EtOH extract of C. *sativa* (at 190 µg/mL) had a significant impact on reducing the production of IL-8, matrix metallopeptidase 9, and cyclooxygenase-2. The same effect was observed when the extract and fraction were combined. The authors also assessed the anti-inflammatory effects of pure CBD and found that it only exerted anti-inflammatory effects at low dosages (16 µg/mL) and not in higher doses (252 µg/mL) in HCT116 colon cancer cells. Since the THCA-rich fraction exhibited anti-inflammatory effects in a wide range of dosages, contrary to pure CBD, it can be concluded that THCA-enriched fractions have a higher anti-inflammatory potential in CRC cell lines. In this sense, combinations of fractions rich in different cannabinoids might have higher pharmacological potential than pure compounds [59]. Conversely, a study by Romano et al. [87] proved that both CBD-rich *C. sativa* extract (SFE-CO_2_) and pure CBD exerted the same antiproliferative effects on CRC DLD-1 and HCT116 cells at 5 µM. This research also identified that the CBD-rich extract had a higher affinity for CB1 and CB2 receptors, compared to pure CBD.

These conflicting findings regarding the effects of cannabinoid-rich extracts and pure compounds may arise from a large number of confounding variables, namely, synergistic interactions, the variability of substance concentrations, the plant’s chemotype, or even the presence of additional bioactive compounds such as terpenes, flavonoids, stilbenoids, and alkaloids. To progress in comprehending the pharmacological activity of *C. sativa* extracts in CRC, it is essential that future studies decipher the impact of each factor.

## 4. Conclusions

Data suggest that cannabinoids exert advantages in the treatment of CRC, mostly by inducing apoptosis, although some evidence also points out that they may target other key therapeutic events, such as proliferation, metastasis, inflammation, angiogenesis, oxidative stress, and autophagy. The currently available data on this subject refer mostly to the *C. sativa* major cannabinoids, i.e., CBD, THC, and CBG, but several pieces of evidence suggest that minor cannabinoids and other bioactive compounds such as terpenes also may hold potential as therapeutic agents for CRC. Data also suggest that certain combinations of cannabinoids and terpenes in *C. sativa* extracts can lead to a synergistic action known as the “entourage effect,” which has been linked to certain pharmacological benefits. The potential therapeutic benefits of the cannabinoids and terpenes from this plant make them key candidates for further drug development.

Even with the shred of evidence for the positive outcomes of the treatment of CRC with *C. sativa*-derived compounds and extracts, several conflicting results can be found across the literature. In addition, the lack of detail on compound interactions and their mechanisms makes it difficult to standardize the treatment of CRC with *C. sativa*, making it imperative to consolidate scientific knowledge in this area.

## Figures and Tables

**Table 1:** Pharmacological effects associated with the major cannabinoids identified in *C. sativa*.

Cannabinoids	Pharmacological Effects	Refs.
CBD	Antiepileptic, antioxidant, anti-inflammatory, antiemetic, immunosuppressive, antipsychotic, neuroprotective, anticancer	[26,32,33,34,35]
Δ^9^-THC	Antioxidant, antipruritic, anti-inflammatory, neuroprotective, analgesic, anticancer, antinausea	[33,34]
CBG	Antibacterial, antifungal, anti-inflammatory, prevents cell proliferation, anticancer, antidepressant, antihypertensive, analgesic	[26,36,37]
CBC	Anti-inflammatory, analgesic	[32,34]
CBN	Sedative, anticonvulsant, anti-inflammatory, antibiotic, anticancer	[32,33,34]
THCV	Weight loss, anticonvulsant, antihyperalgesia, anti-inflammatory, anticancer	[32,33]
CBDV	Inhibits endocannabinoid degradation, antinausea, anticonvulsant, anticancer	[32,33]

CBC, cannabinol; CBD, cannabidiol; CBDV, cannabidivarin; CBG, cannabigerol; CBN, cannabinol; Δ^9^-THC, Δ^9^-tetrahydrocannabinol; THCV, tetrahydrocannabivarin.

**Table 2:** Pharmacological effects of the major terpenes/terpenoids identified in *C. sativa*.

Terpenes/Terpenoids	Pharmacological Effects	Refs.
β-myrcene	Anti-pain, anti-inflammatory, hepatoprotective, analgesic, antioxidant, neuroprotective, gastroprotective, antinociceptive, anticancer, antidiabetic	[32,33,34,43,44]
β/α-caryophyllene	Antibacterial, antifungal, antioxidant, anti-inflammatory, anticancer, anxiolytic	[40,43]
α/β-pinene	Antimetastatic, anti-inflammatory, antibacterial, antidepressant, anticancer	[33,34,40]
D-limonene	Anxiolytic, immunostimulatory, anticancer	[32,34]
Linalool	Sedative, antiepileptic	[34]
Terpineol	Antinociceptive, antifungal, anti-inflammatory, antidiarrheal	[43]
Bisabolol	Anticancer	[33]
β-ocimene	Anticonvulsant, anticancer, antifungal	[26]

**Table 4:** Anti-CRC effects of *C. sativa* extracts rich in cannabinoids and terpenes.

Extraction Method	Bioactive Compounds	Cell Line/Animal Model	Doses	Cancer-Associated Mechanisms	Effects on CRC	Refs.
SFE-CO_2_	CBD (⬆%), Δ^9^-THC, CBG, CBDV, CBDA, CBN	HCT116 and DLD-1 AOM-induced ICR mice Athymic mice (Xen: HCT116)	In vitro: 0.3–5 μM In vivo:10 mg/kg (AOM) 5 mg/kg (Xen)	Proliferation Tumor growth	In vitro: ⬇ proliferation In vivo: ⬇ ACF, polyps, tumor formation, volume	[87]
	ND	CT26 and HCT116 Mice (Xen: CT26)	In vitro: 1, 2, 3, and 4 µg/mL In vivo: 2.5, 5, and 10 mg/kg	Cell survival Proliferation Apoptosis Metastasis	⬇ cell viability (4 µg/mL), colony formation, cyclin D1, CDK4, Bcl-2; ⬆ G0/G1 phase arrest, apoptosis (4 µg/mL), cleaved caspase-3 and PARP	[88]
EtOH extract	⬆% Δ^9^-THC and CBD ⬆% Terpenes	CACO-2 and HT-29	0.24–500 μg/mL	Cell survival	⬆ positive selective cytotoxicity against Ht-29 cells	[42]
	CBG, CBD, CBDA, CBN, CBGA, THC, CBC, and THCA (⬆%)	HCT 116, HT-29, and CACO-2	20 μg/mL (THCA-rich fraction) and 35 μg/mL (CBGA-rich fraction)	Cell survival Apoptosis	⬇ cell viability (EtOH extract, THCA- and CBGA-rich fractions); ⬆ cytotoxicity in THCA-rich fraction combined with EtOH extract fraction, apoptosis (THCA/CBGA-rich fractions), G0/G1 phase arrest (THCA/CBD-rich fractions)	[45]
	CBG, CBD, CBDA, CBN, CBGA, THC, CBC, and THCA (⬆%)	CACO-2, HT-29, HTC116	114–207 μg/mL	Cytotoxicity Inflammation	⬆ cytotoxicity EtOH extract/THCA fraction: ⬇ IL-8, MMP9, COX2	[59]
Maceration (EtOH and MeOH), Soxhlet, UAE (MeOH), SFE CO_2_	CBD, CBDA, THC, THCA, CBGA, CBC, and CBN	CACO-2	0.625–20 μg/mL	Cell survival	Maceration EtOH extract: ⬇ cell viability	[29]

⬆, increase; ⬇, decrease; ACF, aberrant crypt foci; AOM, azoxymethane; Bcl-2, B-cell lymphoma 2; CBD, cannabidiol; CBDA, cannabidiolic acid; CBDV, cannabidivarin; CBG, cannabigerol; CBL, cannabicyclol; CBGV, cannabigerovarin; CBN, cannabinol; CDK4, cyclin-dependent kinase 4; CO_2_, carbon dioxide; COX2, cyclooxygenase-2; DSS, dextran-sodium sulphate; EtOH, ethanol; IL-8, interleukin-8; MeOH, methanol; MMP9, metalloproteinase 9; ND: non-defined; PARP, poly (ADP-ribose) polymerase; SFE-CO_2_, supercritical fluid extraction CO_2_; Δ^9^-THC, Δ^9^-tetrahydrocannabinol; UAE, ultrasound-assisted extraction; XEN, xenograft.

## Data Availability

Not applicable.
